# Relationship Between Estrogen and Idiopathic Mandibular Condylar Resorption: A Systematic Literature Review

**DOI:** 10.3390/medicina61020201

**Published:** 2025-01-23

**Authors:** Benedikta Palesik, Tomas Musulas, Arūnas Vasiliauskas, Dainius Razukevičius, Kristina Lopatienė

**Affiliations:** 1 Department of Orthodontics, Lithuanian University of Health Sciences, LT-50106 Kaunas, Lithuaniaarunas.vasiliauskas@lsmu.lt (A.V.); kristina.lopatiene@lsmu.lt (K.L.); 2 Department of Maxillofacial Surgery, Lithuanian University of Health Sciences, LT-50106 Kaunas, Lithuania; dainius.razukevicius@lsmu.lt

**Keywords:** estrogen, estradiol, idiopathic mandibular condylar resorption, temporomandibular joint disorders, sex hormones, hormones imbalance, bone degeneration, menopause, female hormones, degenerative disease

## Abstract

*Background and Objectives*: Pain in the TMJ is the second most common in the orofacial region. The objective of this systematic review was to assess whether a decrease in estrogen levels increases the risk of idiopathic condylar resorption by reviewing relevant literature and evidence. *Material and Methods*: This systematic review adhered to the Preferred Reporting Items for Systematic Reviews and Meta-Analyses (PRISMA) statement. A comprehensive search was performed in the PubMed (Medline), Science Direct (Elsevier), and Web of Science electronic databases. *Results*: The initial database search identified a total number of 453 studies. After applying the selection criteria, 36 articles were selected for a full-text analysis, and nine studies involving 1105 patients were included in the systematic review. According to the Newcastle–Ottawa Scale (NOS), two of the included articles were graded as being of “Moderate” quality and one was of “Fair” quality. After evaluating the rest of the articles according to the AXIS tool for cross-sectional studies, we generally found that the reliability is moderate. The results show that the decrease in estrogen promotes the occurrence of inflammation in the temporomandibular joint, and some sources mention that it increases the occurrence of idiopathic joint resorption, but we did not establish a complete correlation between the level of estrogen and idiopathic joint resorption. *Conclusions*: This systematic review indicates that there is no evidence suggesting that fluctuations in estrogen levels contribute to idiopathic mandibular condylar resorption, but reduced estrogen levels can be associated with chronic pain in the temporomandibular joint.

## 1. Introduction

Temporomandibular joint disorders (TMDs) involve a range of clinical issues associated with the degeneration of the bone structures and soft tissues of the temporomandibular joint (TMJ) [1,2]. TMJ pain is the second most common type of pain in the orofacial region [2]. The prevalence of TMDs in the global population is 34%, with individuals aged 18 to 60 years being the most susceptible to these disorders [3]. Bone resorption is a process in which cytokine-activated osteoblasts promote osteoclast migration and activity, leading to a progressive decrease in bone volume [4]. The etiology of TMDs is often multifactorial, including orthognathic surgery [5,6], biomechanical factors (occlusal disorders [5,7], bruxism [5,7]), biopsychosocial factors (i.e., anxiety, stress, depression) [8], systemic and local arthritis [5,7], post-traumatic remodeling [5,7], hormonal [5,7] and vitamin imbalance [5,7], infection, vascular necrosis [6], and systemic connective tissue or autoimmune diseases [6]. Idiopathic condylar resorption (ICR) refers to the resorption of the TMJ head of unknown etiology [4]. For treatment planning, it is critical to determine the stage of the patient’s disease and the extent of bone degeneration; therefore, a detailed examination of the patient is required, along with additional blood tests and different X-ray tests [9,10,11]. Detailed anamnesis, physical examination, panoramic radiography, magnetic resonance imaging (MRI), cone beam tomography (CBCT), and computer tomography (CT) are used to determine the exact cause of pain [2,4]. CT and CBCT are used to obtain a better view of bone and bone degeneration, while MRI is commonly used to detect soft tissue abnormalities, including disc positioning [9,10,11]. Blood tests help determine whether the patient has systemic diseases and are tested for rheumatoid arthritis factor, C-reactive protein level, antinuclear antibody level, vitamin D, and 17β-estradiol level [12,13]. The literature mentions that 34% of patients with mild condylar resorption are asymptomatic and do not need treatment [13]. It is also noted that TMD symptoms are much more common in females (in a 2.2:1 ratio) [14]. In every continent, women were found to be diagnosed with TMD at a rate between 9% and 56% [3]. Osteoarthritis is often found in young women (11–15 years old), especially during pubertal growth. Such outcomes are determined by an imbalance of 17 beta-estradiol [15]. Concerning the prevalence of TMDs in females and males, G. Zieliński et al. report a female-to-male ratio (F:M) of 1.56 in South America, 1.26 in North America and Asia, and 1.09 in Europe. These figures suggest a relatively balanced incidence of TMDs between genders across these regions. These findings indicate a relatively proportional incidence of TMDs among both genders across these regions [3].

Estrogen receptors (ERs) are present in TMJ tissues, and modulate inflammatory mediators and cellular responses to mechanical stress [16]. This interaction seems particularly important given the higher prevalence of TMDs in women [16,17]. Estrogens enhance pain responses in TMJ tissues by increasing the release of inflammatory mediators such as cytokines, prostaglandins, and substance P [16]. The release of these mediators is associated with increased pain pathways, suggesting that estrogen may contribute to increased temporomandibular joint pain [16].

Estrogen also stimulates the release of matrix metalloproteinases (MMPs), enzymes that break down collagen and other components of the extracellular matrix, leading to remodeling of the TMJ joints [17]. This structural change can exacerbate TMJ disorders by increasing mechanical stress on the joint, leading to a cycle of pain and inflammation [17].

It has been found that estrogens can affect the trigeminal ganglion, which is involved in the transmission of pain signals from the TMJ to the brain, further intensifying pain sensations in the TMJ area [16].

The aim of this study is to review the articles and determine whether estrogen is associated with idiopathic mandibular condylar resorption.

Hypothesis: The risk of ICR increases with increasing levels of estrogen in the blood.

## 2. Materials and Methods

### 2.1. Study Protocol

This systematic review was registered in the International Prospective Register of Systematic Reviews (PROSPERO) (registration number: CRD42024557234) and conducted according to the Preferred Reporting Items for Systematic Reviews and Meta-Analyses (PRISMA) guidelines [18].

### 2.2. Focus Question

The focus question was developed based on the Population, Intervention, Comparison, and Outcomes (PICO) design (Table 1) [19].

### 2.3. Search Strategy

A comprehensive search was performed in PubMed (Medline), Science Direct (Elsevier), and Web of Science electronic databases to identify records published from May 1 to 24 August 2024. The search strategy implemented boolean operators (OR, AND) to combine the following keywords and Medical Subject Headings (MeSH) terms: Estrogen [MeSH Terms] OR Oestrogen OR Estradiol [MeSH Terms] OR Estrone [MeSH Terms] OR Gonodal steroid hormones [MeSH Terms] OR Sex hormones OR Hormone replacement therapy [MeSH Terms] OR HRT OR Estrogen replacement therapy [MeSH Terms] OR Polycystic ovary syndrome [MeSH Terms] OR Menopause [MeSH Terms] OR Pregnancy [MeSH Terms] OR Contraceptive agents [MeSH Terms] OR Mandibular condyle [MeSH Terms] OR Condylar resorption OR Temporomandibular joint [MeSH Terms] OR TMJ OR Temporomandibular joint disorders [MeSH Terms] OR TMDs.

### 2.4. Selection of Studies

Two authors (BP and TM) independently examined the search results in two stages. In the initial phase of the study selection process, articles were screened based on their title and abstract. In the second phase, the pre-selected articles were evaluated according to eligibility criteria. Disagreements were resolved by discussion and consultation with the third author (KL).

### 2.5. Inclusion Criteria

- Studies involving human subjects (adults; ≥18 years old) with or without TMJ disorders, specifically, ICR;
- Studies assessing estrogen (estradiol) levels in relation to TMJ disorders, specifically, ICR;
- Research articles published in the preceding 10 years (published between August 2014 and August 2024);
- Prospective studies, retrospective studies, and randomized clinical trials;
- Research articles published in English;
- Full-text articles with open access.

### 2.6. Exclusion Criteria

- Case reports;
- Case series;
- Conference abstracts;
- Reviews and systematic reviews;
- Animal studies;
- In vitro studies;

### 2.7. Data Extraction

The data were collected by one author (TM) using pre-established and customized data extraction tables and analyzed by another author (BP). The extracted data from each study comprised the following: (1) authors/year of study, (2) study design, (3) characteristics of patients (i.e., number, gender, age, study groups), (4) outcomes, and (5) conclusions.

### 2.8. Risk of Bias Assessment

The risk of bias and overall methodological quality of the included studies were independently assessed by two authors (BP and TM). Case-control studies were evaluated using the Newcastle–Ottowa scale (NOS) guidelines [20]. NOS contains three domains: the selection of the study groups (four stars), the comparability of the groups (two stars), and the ascertainment of either the exposure or outcome of interest (three stars). Each domain consists of several items that are assessed using a ‘star system’ to determine the overall risk of bias.

The AXIS tool was used to assess the methodological quality of the cross-sectional studies [21]. The tool consists of 20 questions grouped into key areas (introduction: question no. 1; methods: question no. 2–11; results: question no. 12–16; discussions: question no. 17–18; other: question no. 1–20) to guide the appraisal process.

## 3. Results

### 3.1. Study Selection

**Figure 1:**
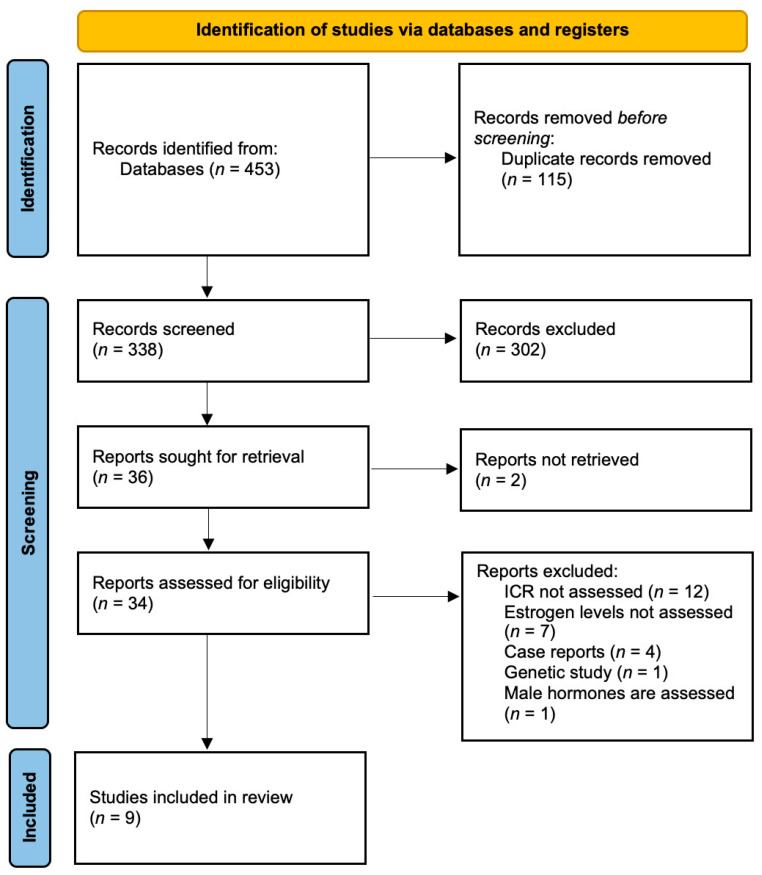
PRISMA flow diagram.

The initial search identified a total of 453 records. After the removal of duplicates, 338 records remained. Independent screening of the titles and abstracts resulted in the selection of 36 articles for a full-text analysis. Nine records that fulfilled the inclusion criteria were included in the systematic review. The detailed process of study selection is provided in the PRISMA flow diagram (Figure 1) [18].

### 3.2. Study Characteristics

A total of nine research studies were included in this systematic review. Of these studies, three were case-control studies [22,23,24], while six incorporated a cross-sectional design [25,26,27,28,29,30]. Table 2 presents data on the characteristics and outcomes of the included studies.

A total of 1105 patients (79 males and 1026 females) were included in the studies [22,23,24,25,26,27,28,29,30]. The average number of patients per study was approximately 123, ranging from a minimum of 18 to a maximum of 353 patients. The age range of patients across the studies varied, with most authors focusing on participants within the 18 to 40 years age range.

All included studies [22,23,24,25,26,27,28,29,30] received approval from their ethical committees or review boards, and the procedures were carried out in accordance with ethical guidelines.

The presence of TMDs was assessed using the Research Diagnostic Criteria for Temporomandibular Disorders (RDC/TMD) [23,24,25,27,28,30] and Okeson’s Muscle and TMJ Examination and Treatment Outcome Form [26]. The TMJ pain was recorded by using the Visual Analogue Scale [26,27,28] and Graded Chronic Pain Scale [25,29,30]. The blood samples were analyzed in four studies [23,24,25,30]. The researchers also used the Patient Health Questionnaire-9 (PHQ-9), Patient Health Questionnaire-15 (PHQ-15), and Generalized Anxiety Disorder-7 (GAD-7) scale [29].

All nine studies analyzed the relationship between TMDs and estrogen changes during different menstrual cycles and related disorders [22,23,24,25,26,27,28,29,30]. Two studies aimed to investigate the association between TMDs and polycystic ovary syndrome (PCOS), with a particular focus on the potential influence of systemic mediators and sex hormones in TMD pathogenesis [23,26]. Ivković et al. and Yuan et al. aimed to determine whether serum estrogen levels are linked to chronic pain, masticatory dysfunction, depressive symptoms, and/or somatization in women with TMDs and varying menstrual cycle statuses [22,25]. One study investigated the association between TMDs, monocytic hyperinflammatory responses, and clinical pain, analyzing whether women with TMDs exhibit a monocytic hyperinflammatory response compared with control women [24]. Vilanova et al. evaluated the associations between hormonal fluctuations during the menstrual cycle, pain levels, maximum occlusal force, and masticatory performance in women with TMDs [27]. A study by Lora et. al. aimed to investigate the prevalence of TMDs in postmenopausal women and the relationship with pain and hormone replacement therapy (HRT) [28]. Minervini et al. focused on evaluating the influence of specific pregnancy-related factors on the prevalence and severity of TMDs [29]. A study by Jedynak et al. aimed to assess the types and prevalence of TMDs in women of reproductive age with menstrual disorders [30].

### 3.3. Quality Assessment

The results of the risk of bias assessment for case-control studies are presented below in Table 3. Using the NOS tool, two studies were evaluated as being of moderate quality (medium risk of bias) [23,24]; one study can be considered as being of high methodological quality (low risk of bias) [22]. Table 4 presents the quality assessment of cross-sectional studies. Using the AXIS questionnaire to evaluate biases, all six cross-sectional studies were considered to be of moderate quality [25,26,27,28,29,30]. Considering that the majority of the included studies were identified as having a moderate risk of bias, the findings require cautious interpretation.

## 4. Discussion

Condylar resorption is a serious degenerative disease of the TMJ bony structures that occurs when the morphology of the articular heads changes, causing pain and facial asymmetry [4]. Analyzing the articles, a number of authors mention that there is a strong tendency for women to have resorption of TMJ condyles [23,24,25,26,27,28,29,30,31,32]. Research indicates that female sex hormones, particularly estrogens, have a crucial role in controlling the metabolism of disc cartilage, bone, and soft tissue in the TMJ [33]. The impact of systemic disorders on the prevalence of aggressive TMJ articular head resorption is the least examined. The impact of estrogens on inflammation is profound. Nonetheless, regarding condylar resorption, the relevant pathways associated with reduced 17β-estradiol involve intercellular mechanisms, including cytokine-nuclear factor-κB ligand (RANKL). These two different cytokines support the integrity of bone tissue. Osteoblasts, T cells, and synoviocytes synthesize these cytokines [34,35]. RANKL has been shown to increase bone resorption through the activation of osteoclasts. Researchers have discovered that 17β-estradiol facilitates osteoprotegerin transcription and inhibits the production of the inflammatory cytokine TNF-α. This protects bones from both local and systemic inflammatory factors [36,37].

In the absence of 17β-estradiol, osteoprotegerin is not activated, resulting in local and systemic inflammatory factors inhibiting new bone tissue development and facilitating bone resorption [36,37]. According to Hatcher’s study, soft tissue changes occur first before condyle resorption. Initially, an irreversible displacement of the disc occurs, and then patients experience limited dislocation and pain, initiating the first stage of TMJ condyle destruction. The sequence of events is assumed to proceed as follows: Initially, cortical bone is resorbed along the anterosuperior surface of the condyles, leading to a defect that penetrates the subchondral bone, resulting in the complete loss of condylar volume [38].

Abubaker et al. discovered that women exhibiting joint complaints were five times more likely to possess elevated levels of intracapsular estrogen receptors compared to those without TMJ concerns [39]. Zielinski and other authors suggest that estrogen levels play an important role in modulating pain, affecting pain perception and sensitivity [16]. Research suggests that fluctuations in estrogen, particularly during phases of the reproductive cycle, can alter the brain’s processing of pain, making people more sensitive to pain when estrogen levels are low [16]. This relationship between estrogen and pain may explain some gender differences in the experience of pain and help guide new pain management strategies [16]. However, according to these studies [16,17], there is insufficient evidence to either justify or contradict the impact of estrogen on the prevalence of TMDs.

The studies [22,23,24,25,26,27,28,29,30] included in this systematic review provide essential insights into the complex association between hormonal fluctuations and TMDs. Particular focus has been placed on gender-specific factors, including pregnancy, PCOS, and menopause, which significantly affect the prevalence and severity of TMDs.

The increased prevalence of TMJ disorders in women may be associated with physiological hormonal fluctuations; however, additional factors might have an impact. The production of relaxin, which is mainly observed during pregnancy, results in generalized joint hypermobility and may contribute to the development of TMDs [40]. Minervini et al. established significant associations between psychosomatic and psychological symptoms and factors such as age and pregnancy trimester in pregnant women, emphasizing the importance of assessing the timing of pregnancy and addressing psychological well-being in the management of TMDs [29]. An epidemiological survey conducted by Fichera G. et al. suggests that the increase in plasma levels of specific female hormones during pregnancy may elevate the risk of dysfunctional TMJ signs and symptoms in pregnant women [31]. Ivković et al. aimed to assess the association between serum estrogen levels and chronic pain, masticatory dysfunction, depressive symptoms, and/or somatization in women with TMDs across varying menstrual cycle status. This study concludes that TMD-related chronic pain severity, masticatory dysfunction, depressive symptoms, and somatization occur most frequently at the lowest estrogen levels, suggesting that surgical menopause plays an important role in the development of TMDs [25]. Genetic factors have a significant impact on the connection between hormones and TMDs. Quinelato et al. identified alterations in the ESR1 and ESRRB genes as increasing the risk of developing TMDs associated with chronic joint pain, pointing to a genetic predisposition that may interact with hormonal changes [32].

Furthermore, a study by Yuan et al. shows that an irregular menstrual cycle and the use of oral contraceptives might not comprehend the initiation and progression of ICR [15]. Although the study sample included participants of both genders, the results indicate that elevated levels of circulating testosterone have been linked with the pathogenesis of ICR in male patients. In addition, the analysis revealed no differences in serum E2 levels or other sex hormone levels between female ICR and disc displacement patients.

The objective of Lora et al. was to investigate the prevalence of TMDs in postmenopausal women and its correlation with pain and HRT, resulting in findings suggesting no association between the use of HRT between the presence of TMDs and the pain threshold [28]. Another study analyzed the hormonal impact on pain perception during the menstrual cycle [40], leading to conclusions that the patients with TMDs exhibited no difference throughout the menstrual cycle, indicating that chronic pain is probably more associated with psychosocial factors than hormonal changes. In the study by Jedynak et al., most patients were hospitalized due to hypothalamic pituitarism (group II of menstrual disorders acc. to World Health Organization classification). According to the results, a statistically significant association was found between masticatory disfunction and group II of menstrual disorders (*p* = 0.0001). In general, the investigation concludes that the development of TMDs may be linked to menstrual disorders [30].

Moreover, psychological factors are linked to the development of TMDs [8]. Psychosocial illnesses, such as anxiety and depression, are acknowledged for being associated with TMDs. TMD-related pain might, on the other hand, induce psychological problems. In addition, psychological stress may negatively affect patient compliance, altering their awareness of the disorder and diminishing their motivation for treatment [8].

To ensure that the most recent evidence on the association between estrogen (estradiol) levels and TMDs, specifically, ICR, was included, studies from the last 10 years were selected. Over the past decade, improvements in diagnostic techniques, updated clinical criteria, and a greater awareness of hormonal influences have produced more precise and relevant data for a comprehensive review. Including older studies could introduce outdated methodologies or inconsistent criteria, potentially limiting the reliability and relevance of our findings.

The findings of this systematic review are limited by the lack of investigations specifically focused on the relationship between hormonal changes and idiopathic mandibular condylar resorption. Some authors mentioned that the sample size was small [24,25,28,29]. The studies [24,25,28,30] did not assess the impact of high physiological estrogen levels or other phases of the menstrual cycle on TMJ. The study by Yazici et al. had certain limitations, including the lack of assessment of local levels of sex hormones, inflammatory mediators, and MMPs [23]. Local rather than systemic assessment of these factors might have yielded a clearer understanding of the mechanisms behind TMD development in PCOS. Ribeiro-Dasilva et al. acknowledge that inflammation is a well-established contributor to arthrogenous TMD, but its role in myogenous pain remains less understood. Due to the small sample size, the researchers assessed only one aspect of inflammation, the receptor (estrogen), and a single response mediator, the toll-like receptor (TLR)-4 ligand. Consequently, further studies with other factors that contribute to the development of TMD should be performed [24]. Ivković with co-authors [25] analyzed three groups of patients: (1) women with a normal menstrual cycle, (2) pregnant women, and (3) postmenopausal women. However, the age differences between these groups may have influenced the results. To obtain more accurate research data, studies should be conducted in one age group or to compare how estrogen levels and TMD symptoms change with age. Lora et al. analyzed the correlation between hormone use and TMD. However, in a random sample of TMD cases identified in the community, associations between hormone use and TMD might not have been observed, which would be a limitation of the study [28]. Minervini et al. [29] used a questionnaire; therefore, the responses may have been influenced by subjective bias, as they depended on participants’ memories and perceptions, which could vary based on individual differences and mood when completing the questionnaire. In the study by Jedynak et al., the diagnosis of TMD was based only on the DC/TMD criteria [30]. Additional investigations are necessary to draw more definitive conclusions and ultimately enhance patient management approaches.

## 5. Conclusions

This systematic review indicates that there is no evidence suggesting that fluctuations in estrogen levels contribute to idiopathic mandibular condylar resorption. Reduced estrogen levels can be associated with chronic pain in the temporomandibular joint, muscle tenderness, and masticatory dysfunction.

## Figures and Tables

**Table 1:** PICO design.

Element	Inclusion
(P) Population	Adults (≥18 years old), females and males, diagnosed with TMDs (specifically ICR), and with estrogen (estradiol) levels recorded
(I) Intervention	Investigation of TMDs (specifically ICR) and estrogen (estradiol) levels
(C) Comparison	TMD (specifically ICR) patients and healthy control groups; comparison within TMD (specifically ICR) patients with and without estrogen (estradiol) related conditions
(O) Outcomes	Impact of estrogen level on the occurrence, severity, and progression of TMDs (specifically ICR)

TMD, temporomandibular joint disorder; ICR, idiopathic condylar resorption.

**Table 2:** Characteristics of included studies.

Authors (Year),	Study Design	Patients (M/F), Mean Age (SD)	Outcomes	Conclusions
Yuan et al. (2021) [22]	Case-control	94 (14/80) with ICR, mean age 20.9 (SD not defined) (range 10.2–39.8 years); 259 (65/259) with DD, mean age 20.2 (SD not defined) (range 10.6–42.7 years).	FSH ICR: 4.99 ± 1.80 IU/L; ICR (female): 5.21 ± 1.59 IU/L; ICR (male): 3.70 ± 2.41 IU/L; DD: 4.89 ± 2.29 IU/L; DD (female): 5.34 ± 2.11 IU/L; DD (male): 3.11 ± 2.13 IU/L. LH ICR: 3.92 ± 2.86 IU/L; ICR (female): 3.99 ± 2.89 IU/L; ICR (male): 3.51 ± 2.73 IU/L; DD: 3.91 ± 2.06 IU/L; DD (female): 4.26 ± 3.20 IU/L; DD (male): 2.52 ± 1.86 IU/L. PRL ICR: 15.19 ± 21.37 ng/mL; ICR (female): 15.55 ± 22.07 ng/mL; ICR (male): 13.11 ± 17.34 ng/mL; DD: 12.05 ± 7.17 ng/mL; DD (female): 12.43 ± 7.52 ng/mL; DD (male): 10.56 ± 5.40 ng/mL. E2 ICR: 35.59 ± 18.94 pg/mL; ICR (female): 36.93 ± 19.67 pg/mL; ICR (male): 27.93 ± 11.88 pg/mL; DD: 32.09 ± 14.20 pg/mL; DD (female): 34.36 ± 14.31 pg/mL; DD (male): 23.03 ± 9.35 pg/mL. Testosterone ICR: 1.59 ± 4.53 ng/dL; ICR (female): 0.74 ± 4.13 ng/dL; ICR (male): 6.41 ± 3.65 ng/dL; DD: 1.03 ± 1.79 ng/dL; DD (female): 0.27 ± 0.11 ng/dL; DD (male): 4.08 ± 2.08 ng/dL. Progesterone ICR: 0.33 ± 0.74 ng/mL; ICR (female): 0.35 ± 0.80 ng/mL; ICR (male): 0.23 ± 0.09 ng/mL; DD: 0.27 ± 0.26 ng/mL; DD (female): 0.29 ± 0.27 ng/mL; DD (male): 0.20 ± 0.18 ng/mL.	No differences in sex hormone levels were observed between female ICR and DD patients. Increased testosterone levels were linked to the pathogenesis of ICR in male patients.
Yazici et al. (2020) [23]	Case-control	45 females with PCOS, mean age 27.8 (6.5); Control: 30 females, mean age 30.4 (5.1).	Incidence of TMDs PCOS: 51.5%; Control: 6.9%. Progesterone levels PCOS: 1.3 ± 0.80 ng/L; Control: 4.07 ± 0.85. PCOS patients with TMDs: 0.61 ± 0.29 ng/L; PCOS patients without TMDs: 2.03 ± 0.43 ng/L. Estrogen levels PCOS: 47.6 ± 40.3 pg/mL; Control: 44.3 ± 41.5 pg/mL; PCOS patients with TMDs: 51.9 ± 66.2 pg/mL; PCOS patients without TMDs: 42.8 ± 23.4 pg/ml.	TMDs incidence was significantly higher in PCOS patients. The PCOS group exhibited significantly lower midluteal progesterone levels than the control group. No differences in estrogen levels were noted between PCOS patients with TMDs and those without TMDs.
Ivković et al. (2018) [25]	Cross-sectional	67 females Group 1: 28 (normal menstrual cycle), mean age 24.08 (4.0); Group 2: 23 (pregnant), mean age 27.75 (4.7); Group 3: 13 (surgical menopause), mean age 40.56 (2.8).	Chronic pain degree G1 (normal menstrual cycle): (0): 7; (I) 8; (II): 8; (III) 4; (IV): 1. G2 (pregnant): 4 weeks (0): 7; (I) 7; (II): 6; (III) 2; (IV): 1; 12 weeks (0): 13; (I) 6; (II): 4; (III) 0; (IV): 0; 24 weeks (0): 17; (I) 5; (II): 1; (III) 0; (IV): 0; 36 weeks (0): 23; (I) 0; (II): 0; (III) 0; (IV): 0. G3 (surgical menopause): (0): 2; (I) 4; (II): 3; (III) 2; (IV): 2; Depressive symptoms G1: (none): 13; (moderate): 9; (severe): 6. G2: 4 weeks (none): 10; (moderate): 8; (severe): 5; 12 weeks (none): 12; (moderate): 8; (severe): 3; 24 weeks (none): 15; (moderate): 7; (severe): 1; 36 weeks (none): 20; (moderate): 3; (severe): 0. G3: (none): 2; (moderate): 5; (severe): 6. Somatization G1: (none): 14; (moderate): 9; (severe): 5. G2: 4 weeks (none): 9; (moderate): 7; (severe): 7; 12 weeks (none): 16; (moderate): 4; (severe): 3; 24 weeks (none): 18; (moderate): 4; (severe): 1; 36 weeks (none): 21; (moderate): 2; (severe): 0. G3: (none): 2; (moderate): 4; (severe): 7. Limitation in masticatory function Chewing G1: (yes): 10; (no): 18; G2: (yes): 6; (no): 17; G3: (yes): 9; (no): 4. Drinking G1: (yes): 8; (no): 20; G2: (yes): 5; (no): 18; G3: (yes): 3; (no): 10. Eating hard food G1: (yes): 9; (no): 19; G2: (yes): 6; (no): 17; G3: (yes): 7; (no): 6. Eating soft food G1: (yes): 2; (no): 26; G2: (yes): 6; (no): 17; G3: (yes): 5; (no): 8. Smiling/laughing G1: (yes): 1; (no): 27; G2: (yes): 2; (no): 21; G3: (yes): 0; (no): 13. Yawning G1: (yes): 3; (no): 25; G2: (yes): 3; (no): 20; G3: (yes): 0; (no): 13. Swallowing G1: (yes): 3; (no): 25; G2: (yes): 0; (no): 23; G3: (yes): 0; (no): 13. Talking G1: (yes): 2; (no): 26; G2: (yes): 0; (no): 23; G3: (yes): 0; (no): 13.	Reduced estrogen levels can be associated with chronic pain linked to TMDs, masticatory dysfunction, depressive symptoms, and somatization.
Soydan et al. (2014) [26]	Cross-sectional	50 females with PCOS, mean age 27 (6); Control: 50 healthy females, mean age 26 (5).	Incidence of TMDs PCOS: 43 (86); Control: 12 (24). Incidence of TMJ pain PCOS: 36 (72); Control: 14 (28). VAS PCOS: 2.9 ± 2.61; Control: 0.3 ± 1.56. Incidence of muscular tenderness PCOS: 32 (64); Control: 14 (24).	The incidence of TMDs, muscle tenderness, and pain in the TMJ were significantly higher in patients with PCOS.
Ribeiro-Dasilva et al. (2017) [24]	Case-control	9 females with TMDs, mean age 26 (7); Control: 9 females, mean age 25 (7).	Pain characteristics (values on a scale of 0 to 10) TMD patients: Worst pain in last 6 months: 7.33 ± 2.062; Average pain in last 6 months: 5.11 ± 2.369; Pain interference in the last 6 months: 3.89 ± 3.00; Pain interference with daily activity: 8.56 ± 2.00; Pain interference with work activity: 2.00 ± 1.00; Pain interference with social life: 2.00 ± 1.00; Control: N/A.	Increased monocytic responses to estrogen were associated with elevated pain levels in TMDs patients.
Vilanova et al. (2015) [27]	Cross-sectional	MC group: 25 females, mean age 24.7 (6.2); OC group: 25 females, mean age 29.2 (7.4).	Pain level (mm) OC 1st assessment: 3.8 ± 4.9; OC 2nd assessment: 3.0 ± 4.0; OC 3rd assessment: 2.0 ± 2.8; OC 4th assessment: 3.4 ± 4.5; MC (menstrual): 3.6 ± 4.6; MC (follicular): 3.5 ± 4.6; MC (ovulatory): 2.9 ± 3.8; MC (luteal): 3.6 ± 4.8. Maximum occlusal force (KgF) OC: 39.59 ± 10.62; MC: 46.52 ± 11.09.	Hormonal fluctuations throughout the menstrual cycle influenced pain levels in women with TMDs without affecting their masticatory function.
Lora et al. (2016) [28]	Cross-sectional	155 postmenopausal females without TMDs; 129 postmenopausal females with TMDs, mean age 56.7 (SD not defined).	No/low pain Postmenopausal females with TMDs: 94 (72.87); Postmenopausal females without TMDs: 155 (100). Moderate/severe pain Postmenopausal females with TMDs: 35 (27.13). HRT Postmenopausal females without TMDs: (no): 113 (72.90); (yes): 42 (27.10); Postmenopausal females with TMDs (without pain): (no): 68 (72.34); (yes): 26 (27.66); Postmenopausal females with TMDs (with pain): (no): 15 (42.86); (yes): 20 (57.14).	No association between HRT and the prevalence and severity of TMDs in postmenopausal women was observed.
Minervini et al. (2024) [29]	Cross-sectional	32 pregnant females, mean age and SD not defined (age range 18–50 years).	Somatic symptom severity (PHQ15) Data were available only in graphical form; exact numerical values were not provided.	Hormonal changes are associated with an increased prevalence and severity of TMDs in pregnant women.
Jedynak et al. (2021) [30]	Cross-sectional	65 females with menstrual/hormonal disorders, mean age 28 (6.27); Control: 61 age-gender matched healthy females.	TMDs Hormonal disorders: 60 (92.3); Control: 34 (55.73). MFP Hormonal disorders: 29 (44.62); Control: 24 (39.34). DDwR Hormonal disorders: 30 (46.15); Control: 9 (14.75). DDw/oR Hormonal disorders: 11 (16.92); Control: 2 (3.28). DJD Hormonal disorders: 8 (12.3); Control: 3 (4.92).	Hormonal fluctuations and menstrual cycle irregularities can be associated with the development or exacerbation of TMDs.

ICR, idiopathic condylar resorption; DD, disc displacement; FSH, follicle-stimulating hormone; LH, luteinizing hormone; PRL, prolactin; E2, 17β-estradiol; PCOS, polycystic ovary syndrome; TMJ, temporomandibular joint; VAS, visual analogue scale; TMDs, temporomandibular joint disorders; MC, menstrual cycle; OC, oral contraceptive; HRT, hormonal replacement therapy; MFP, myofascial pain; DDwR, articular disc displacement with reduction; DDw/oR, articular disc displacement without reduction; DJD, temporomandibular joint degeneration.

**Table 3:** Risk of bias assessment using Newcastle–Ottawa scale for case-control studies.

Authors (Year)	Selection 0–4	Comparability 0–2	Outcome 0–3	Score (Risk of Bias)
Yuan et al. (2021) [22]	**★★★★**	**★**	**★★**	7/9 (Low)
Yazici et al. (2020) [23]	**★★**	**★**	**★★**	5/9 (Moderate)
Ribeiro-Dasilva et al. (2017) [24]	**★★**	**★**	**★★**	5/9 (Moderate)

(No. of **★**): 7–9 (low risk of bias); 4–6 (moderate risk of bias); 0–3 (high risk of bias).

**Table 4:** Risk of bias assessment using AXIS tool for cross-sectional studies.

Authors (Year)	Question No.																				Overall Appraisal
	1	2	3	4	5	6	7	8	9	10	11	12	13	14	15	16	17	18	19	20	
Ivković et al. (2018) [25]	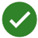	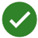	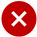	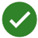	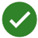	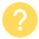	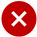	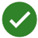	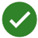	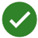	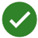	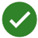	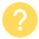	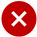	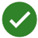	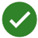	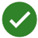	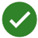	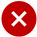	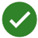	Moderate
Soydan et al. (2014) [26]	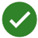	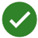	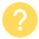	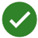	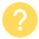	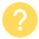	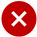	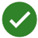	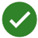	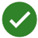	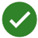	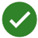	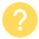	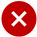	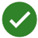	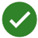	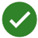	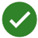	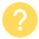	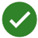	
Vilanova et al. (2015) [27]	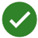	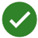	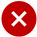	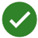	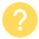	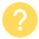	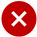	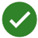	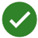	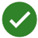	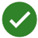	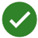	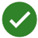	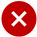	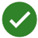	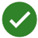	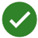	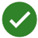	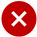	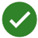	
Lora et al. (2016) [28]	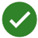	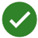	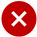	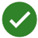	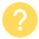	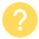	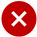	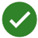	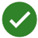	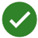	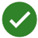	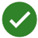	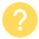	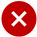	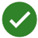	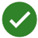	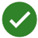	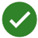	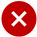	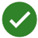	
Minervini et al. (2024) [29]	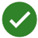	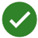	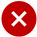	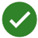	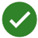	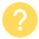	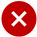	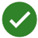	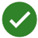	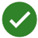	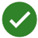	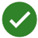	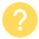	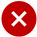	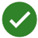	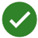	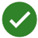	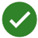	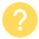	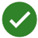	
Jedynak et al. (2021) [30]	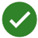	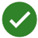	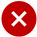	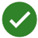	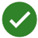	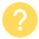	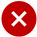	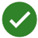	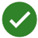	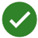	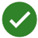	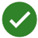	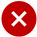	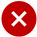	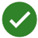	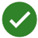	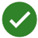	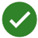	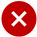	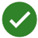	

Questions: 1. Were the aims/objectives of the study clear? 2. Was the study design appropriate for the stated aim(s)? 3. Was the sample size justified? 4. Was the target/reference population clearly defined? (Is it clear who the research was about?) 5. Was the sample frame taken from an appropriate population base so that it closely represented the target/reference population under investigation? 6. Was the selection process likely to select subjects/participants that were representative of the target/reference population under investigation? 7. Were measures undertaken to address and categorize non-responders? 8. Were the risk factor and outcome variables measured appropriate to the aims of the study? 9. Were the risk factor and outcome variables measured correctly using instruments/measurements that had been trialed, piloted, or published previously? 10. Is it clear what was used to determined statistical significance and/or precision estimates (e.g., *p*-values, confidence intervals)? 11. Were the methods (including statistical methods) sufficiently described to enable them to be repeated? 12. Were the basic data adequately described? 13. Does the response rate raise concerns about non-response bias? 14. If appropriate, was information about non-responders described? 15. Were the results internally consistent? 16. Were the results for the analyses described in the methods, presented? 17. Were the authors’ discussions and conclusions justified by the results? 18. Were the limitations of the study discussed? 19. Were there any funding sources or conflicts of interest that may affect the authors’ interpretation of the results? 20. Was ethical approval or consent of participants attained? 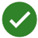 yes; 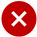 no; 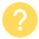 unclear.
